# The Efficiency of Extracorporeal Shock Wave Lithotripsy (ESWL) in the Treatment of Distal Ureteral Stones: An Unjustly Forgotten Option?

**DOI:** 10.7759/cureus.28671

**Published:** 2022-09-01

**Authors:** Jasmin Alić, Jasmina Heljić, Osman Hadžiosmanović, Benjamin Kulovac, Zahid Lepara, Hajrudin Spahović, Senad Bajramović, Damir Aganović

**Affiliations:** 1 Urology, Clinical Center University of Sarajevo, Sarajevo, BIH; 2 Pediatrics, General Hospital "Prim. dr Abdulah Nakaš", Sarajevo, BIH; 3 Urology and Reconstructive Surgery, Sarajevo School of Science and Technology, Sarajevo, BIH

**Keywords:** treatment efficiency, distal ureter, ureteral stone, eswl, extracorporeal shock wave lithotripsy

## Abstract

Introduction

The optimal management of distal ureteral stones remains a matter of debate since current guidelines favor ureteroscopy over extracorporeal shock wave lithotripsy (ESWL). We aimed to evaluate the efficiency of ESWL for distal ureteral stones and to identify factors that affect treatment outcomes.

Materials and methods

The retrospective study included records of 115 patients with distal ureteral stones, 5 mm to 18 mm in size, undergoing 223 ESWL sessions as an outpatient procedure. Early fragmentation and three-month follow-up stone-free rate (SFR) was assessed through radiographic imaging. Treatment was successful if there were no residual fragments or they were ≤4 mm, three months after the last session.

Results

The mean ±standard deviation (range) stone size was 9.68 ±3.10 (5.00-18.0) mm. The mean body mass index (BMI) was 24.3 ±2.67 (18.4-29.8) kg/m² with a significant correlation between BMI and stone size (r^2 ^=0.324, p <0.001). Patients underwent ESWL an average of 1.7 ±1.36 times (1-5), while 68 patients (59.1%) became stone-free after one session. The overall SFR was 82.6%; for patients with stone sizes ≤10 mm and >10 mm, it was 99% and 9.4%, respectively. Cumulative SFR after the second session was 77%. In 20 (17%) patients the treatment was a failure. Complications occurred in 10.4%, while auxiliary procedures were needed in 8.7% of cases, both significantly affected by the stone size (p <0.001). The efficiency quotient (EQ) was 0.76. Treatment outcome was significantly different depending on stone size, BMI, number of sessions, complications, and auxiliary procedures (p <0.001, p =0.022, p <0.001, p <0.001, p <0.001, respectively). Univariate regression analysis identified stone size and BMI as significant predictors of treatment outcome (odds ratio (OR) 3.84, 95% confidence interval (CI): 2.31-8.97, p =0.001, and OR 1.25, 95% CI: 1.04-1.54, p =0.024, respectively).

Conclusions

Extracorporeal shock wave lithotripsy continues to be a safe and effective option for managing simple calculi in distal ureters with a diameter of ≤10 mm. The stone size and BMI remain significant predictors of treatment outcome.

## Introduction

Urinary stones are among the most common urological conditions that affect millions worldwide and place a significant burden on the healthcare system [1]. It is found in approximately one in 11 people in Western countries [2]. Ureteral stones account for 20% of urinary stones with up to 70% localized in the distal ureter [3,4].

Treatment depends on size, position, composition, clinical factors, equipment availability, and urologist experience [5]. In the era of improvement of endoscopic procedures, optimal management of distal ureteral stones remains a matter of debate. Current guidelines recommend extracorporeal shock wave lithotripsy (ESWL) as a secondary option as ureteroscopy (URS) was found to be superior in the treatment of lower urinary stones [6-8].

Extracorporeal shock wave lithotripsy is well established as safe, non-invasive, highly efficient, and more cost-effective than endoscopic interventions, which is why it has been introduced as a preferred treatment modality worldwide [5,8]. Although ESWL has a lower complication rate and shorter hospital lengths, overall, it has lower stone-free rates (SFRs) [9]. In addition, ureteroscopy with holmium laser lithotripsy was found to be more efficient with a shorter operative time [10]. However, the global pandemic of severe acute respiratory syndrome coronavirus 2 (SARS-COV-2) may lead to a further increase in ESWL use as it does not require general anesthesia and thus avoids its potential complications in patients with COVID-19 [11].

Extracorporeal shock wave lithotripsy uses generated acoustic waves which damage stones due to the effects of direct stresses and cavitation. Despite its significant benefits, adverse effects of ESWL have been described, as concomitant tissue injury occurs [12]. Equipment and technique have been refined over the decades in trying to minimize postoperative recovery and morbidity [5].

Spontaneous elimination for distal ureteral stones 5 mm to 10 mm in size amounts to 25% to 50% or even higher during the prolonged follow-up period [13]. The ESWL success rate is 82% to 90% and 58% to 67% for proximal/mid and distal ureteral stones, respectively [14].

The efficiency of ESWL measured by success rate is affected by stone and patient-related factors, which include stone size, position, degree of impaction, ureter anatomy, skin-to-stone distance (SSD), stone density measured in Hounsfield units (HU), habitus, body mass index (BMI), and the presence of a ureteral stent [5]. In addition, lithotripter performances, shock wave delivery rate, and power modifications, together with imaging technology and service setup are among the most significant determinants of ESWL success [5,15]. Furthermore, the adjuvant use of pharmacological agents, such as alpha-blockers and/or potassium citrate may have an impact on SFR [16]. Appropriate patient selection with planning and post-treatment monitoring is essential for achieving optimal outcomes and utilization of ESWL.

This study aimed to evaluate the efficiency of ESWL for distal ureteral stones regarding age, sex, BMI, stone size, treatment complications, needs for retreatments, and auxiliary procedures and to identify factors that affect treatment outcome.

## Materials and methods

Study design

This retrospective cohort study was performed in a tertiary-level stone center. It included patients with distal ureteral stones undergoing ESWL treatments from May 2014 to November 2021 at the Clinic of Urology, Clinical Center University of Sarajevo in Bosnia & Herzegovina.

Patients

The medical records of 115 patients requiring 223 ESWL sessions were analyzed in the study. The primary selection was performed based on clinical data and radiological findings provided in the records. Patients were included in the study based on specific criteria: unilateral radiopaque stone with the position in the distal ureter, 5 mm to 20 mm in size. Furthermore, it included patients with normal or mildly altered renal function, without previous kidney or ureteral surgery, intravesical obstruction, or proven urinary tract infection. The patients had no history of use of anticoagulants and antihypertensive drugs.

The exclusion criteria were: patients younger than 18 and older than 70 years, BMI >30 kg/m^2^, stone larger than 20 mm, the presence of radiolucent or bilateral ureteral stones, urinary tract infection or systemic inflammation, obstruction and malignancy, patients with a solitary kidney, identified hematoma after ESWL, severe spine deformities, history of tumors, strictures or anatomic anomalies of the ureter and/or previous kidney or ureteral surgery. Also, patients who used additional pharmacological agents such as alpha-blockers were excluded. General exclusion criteria were considered, namely pregnancy, moderate and severely impaired renal function, and patients with pronounced comorbidity such as unstable cardiac status, aortic abdominal aneurysm, renal artery aneurysm, or stenosis.

Equipment

All patients underwent a baseline radiographic evaluation of the kidneys, ureter, and bladder (KUB) and/or an abdominopelvic computed tomography (CT) (non-enhanced or enhanced) scan. The localization and maximum diameter of the stone were determined. However, stone density measured with HU and SSD was not routinely evaluated. Abdominal ultrasonography was used to diagnose obstruction before and after treatment. The distal ureter was defined as the ureteral segment below the lower border of the sacroiliac joint to the vesicoureteral junction.

The ESWL treatment was performed in the supine transgluteal position using a Dornier Compact Delta II unit (Dornier Medizintechnik GmbH, München, Germany), the device with an electromagnetic generation of high-energy shock waves. The voltage of each shock wave was gradually increased from an initial 12 kV to a final voltage of 19 kV. The procedure was ended when satisfactory fragmentation was seen on fluoroscopy or after 4000 shock waves have been delivered at a pulse rate of 84-90/min. During the treatment, the stone was visualized by fluoroscopy and ultrasonography. Extracorporeal shock wave lithotripsy procedure was carried out as an outpatient procedure, without anesthesia and with the application of standard analgesia.

Methods

The medical records contained anamnesis, objective physical examination, laboratory tests, and radiological examination data. The success of ESWL was evaluated according to demographic data, stone size, and position, number of sessions, SFR, complications, and need for auxiliary procedures. The immediate and three-month follow-up SFR were analyzed, as well as the retreatment rate. Successful ESWL treatment was defined as the complete absence of stone fragments in imaging studies (stone-free status (SFS)) or the presence of clinically insignificant residual fragments sized ≤4 mm [17]. Patients requiring repetitive ESWL sessions for the same stone were defined as retreatment.

Patients who had sufficient fragmented stones but were not stone-free were carefully followed and reviewed after seven to 10 days after the initial session using a KUB plain radiograph. Additional ESWL session was applied immediately if there was inadequate stone fragmentation (either no signs of fragmentation or fragments were >4 mm in diameter). Patients who had a significant residual stone (>4 mm) after the third treatment, a residual fragment that failed to pass, and cases in which complications occurred were considered ESWL failure. An alternative treatment modality was suggested for these patients.

A small number of patients required additional procedures, such as ureteral stent placement or ureteroscopy, which were done within 48 hours after the initial procedure if there was a worsening of the symptoms and/or an inability to resolve them conservatively. Patients were evaluated three months after the last ESWL session. No routine antibiotic or medical expulsive treatment (MET) was performed before or after the treatment.

Efficiency quotient (EQ) was assessed using the following equation previously proposed by Denstedt et al. [18]: EQ = % stone free / [100% (1 treatment) + % requiring retreatment + % requiring auxiliary procedure] × 100%.

Statistical analysis

Data were provided as absolute (n) and relative (%) numbers, mean and median values, range, and standard deviation (SD). The Kolmogorov-Smirnov test and Shapiro-Wilk test were used for the data distribution analysis. Statistical analysis was performed using unpaired and paired Welch’s t-test for parametric data and chi-square test and Fisher’s exact test for non-parametric categorical variables. Also, when the number of subjects compared was small, non-parametric Mann-Whitney and Kruskal-Wallis tests were carried out.

The correlation was assessed by Pearson or Spearman method. Univariate analysis was used to assess the association between the individual factors and the ESWL outcome. The significantly associated variables were further tested using multivariate analysis to identify factors that acted independently and to predict the probability of positive or negative treatment outcomes. The specificity and sensitivity of variables were examined by the ROC (receiver operative characteristics) curve. The p-values <0.05 were considered statistically significant. Statistical analysis was performed using Statistical Package for Social Sciences (SPSS) version 23 (IBM Corp., Armonk, NY, USA).

Ethical considerations

This retrospective study obtained the Ethical Board approval (approval number: 29-20-945/12), and met all the local and national ethical requirements, being conducted under the principles described in the Declaration of Helsinki and with good clinical practice protocols. The study was exempt from the written informed consent requirements due to its retrospective design and anonymized patient records.

## Results

The summary of patients and treatment-related characteristics are shown in Table 1. A total of 115 patients with ureteral stones size 5 mm to 18 mm were treated with 223 ESWL sessions. The mean ±SD (range) age was 51.8 ±11.0 (22-68) years with a predominance of male patients (75 (65%) vs. 40 (35%)), and a male/female ratio of 1.87. The stone distribution concerning the side of the body was almost equal. The mean stone size was 9.68 ±3.10 (5.00-18.0) mm. The mean BMI was 24.3 ±2.67 (18.4-29.8) kg/m². There was a statistically significant linear correlation between stone size and BMI (r^2^ =0.324, p <0.001).

**Table 1:** Summary of baseline characteristics Values are presented as absolute, relative numbers, and/or mean ±SD (range) as appropriate. SD: Standard deviation, BMI: Body mass index, SFR: Stone-free rate, n: number

Variable	Value
No. of patients	115
Age (years), mean ±SD (range)	51.8 ±11.0 (22.0-68.0)
BMI (kg/m²), mean ±SD (range)	24.3 ±2.67 (18.4-29.8)
Sex	
male	75 (65%)
female	40 (35%)
Side	
right	58 (50.4%)
left	57 (49.6%)
Stone size (mm), mean ±SD (range)	9.68 ±3.10 (5.00–18.0)
≤10 mm	83 (72.2%)
>10 mm	32 (27.8%)
No. of sessions (n), mean ±SD	223; 1.70 ±1.36
Outcome	
success	95 (83%)
failure	20 (17%)
Auxiliary procedures	
without	105 (91.3%)
ureteral stent	6 (5.2%)
ureteroscopy	4 (3.5%)
Complications	
without	103 (89.57%)
steinstrasse	4 (3.47%)
infection	4 (3.47%)
obstruction	4 (3.47%)
SFR after each session	
1st	68 (59.1%)
2nd	20 (17.4%)
3rd	7 (6.1%)

The mean number of sessions was 1.70 ±1.36 (1-5). The overall success rate was 82.6%. The success rate after the initial and second ESWL sessions was 59.1% and 77%, respectively. Sixty-eight patients (59.1%) became stone-free after one session, while 20 patients (17.4%) needed two, and seven patients (6.1%) needed three sessions (as seen above in Table 1). In 20 patients (17%) the treatment was a failure either due to lack of disintegration or elimination of residual fragments. Separately, in the group of patients with a stone size ≤10 mm, the success rate after three months was 99%. The analysis showed a statistically significant difference between stone size and the number of sessions only (p <0.001). Simultaneously, there was a statistically significant linear correlation between stone size and BMI in this group (r^2^ =0.340, p <0.01).

In the group of patients with a stone size >10 mm, there was a statistically significant difference between stone size and treatment outcome (p <0.001), same as the stone size and auxiliary procedures (p <0.001). Also, there was a statistically significant correlation between stone size and patient's age (r^2^= -0.374, p <0.035) and stone size and the number of sessions (r^2^= -0.609, p <0.001). The mean number of sessions in this group was 2.84 ±1.02 (1-5) and the success rate after three months was 9.38%.

Auxiliary procedures were needed in 10 cases (8.7%) and they included ureteral stent placement in six cases (5.2%) and ureteroscopy with stone extraction in four cases (3.5%). Although stent placement was followed by repeated successful ESWL sessions, these cases were considered a failure (as seen above in Table 1). Nine patients who had poor fragmentation after three ESWL sessions refused to undergo salvage ureteroscopy, but they achieved SFS after multiple sessions, although these cases too were considered a failure. The analysis showed that auxiliary procedures were significantly different depending on stone size, BMI, the number of sessions, and complications (p <0.001, p=0.042, p <0.001, p=<0.01, respectively).

Treatment complications were detected in 16 patients (10.4%); four developed distal steinstrasse, which was resolved conservatively or by additional ESWL treatment of leading fragment; four patients each developed urinary tract infection and urinary tract obstruction, which were resolved conservatively or by the placement of the ureteral stent and/or salvage URS. Ureteric colic was reported by most of the patients and was managed conservatively. Given all these data, and using the formula above, the EQ was 0.76.

Treatment complications were significantly different depending on stone size, auxiliary procedures, and number of sessions (p <0.001, p <0.01, p <0.001, respectively) as their number and severity increased with increasing these values. However, the complications are not significantly different depending on the BMI, age, and body side (p=0.097, p=0.47, p=0.94, respectively).

**Figure 1:**
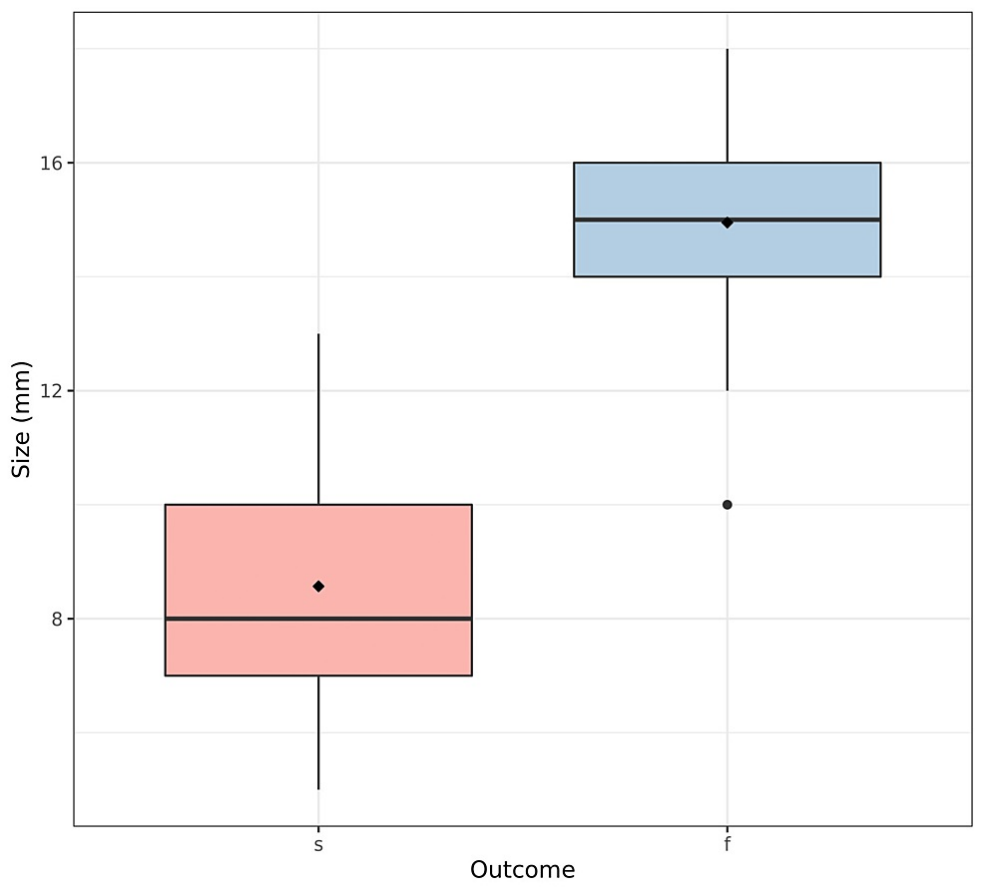
Treatment outcome according to stone size s: Success, f: Failure

**Figure 2:**
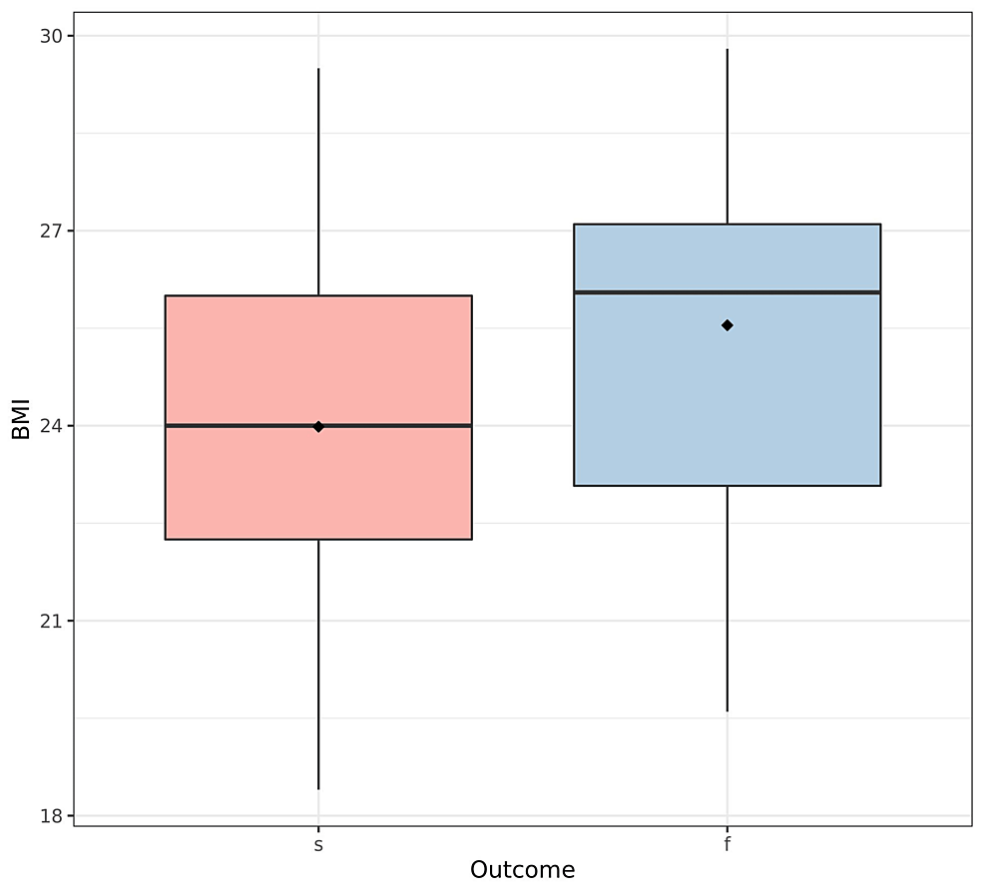
Treatment outcome according to BMI (kg/m²) BMI: Body mass index, s: Success, f: Failure

**Figure 3:**
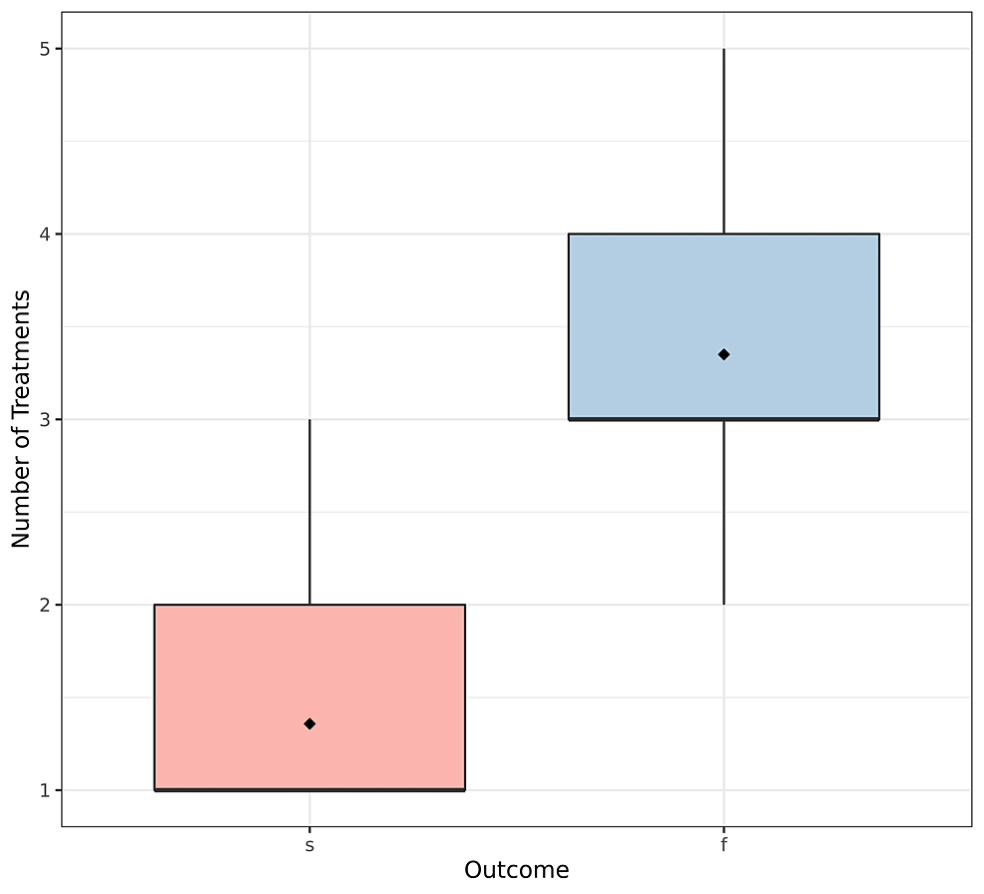
Treatment outcome according to number of sessions s: Success, f: Failure

The explanatory analysis showed that stone size, BMI, number of sessions, distribution of complications, and auxiliary procedures were significantly different depending on treatment outcome (p <0.001, p=0.022, p <0.001, p <0.001, p <0.001, respectively) (Figures 1-3). However, the side distribution, sex, and age were not significantly different depending on ESWL outcome (p=0.97, p=0.98, p=0.92, respectively) (Table 2).

**Table 2:** Univariate analysis of the variables affecting the outcome of ESWL treatment Values are presented as absolute, relative numbers, mean (SD, range), and/or median (Q25-75) as appropriate. ESWL: Extracorporeal shock wave lithotripsy, n: Number, SD: Standard deviation

		Success (n=95)	Failure (n=20)	n	p-value	Test
Age (years), mean ±SD		51.9 ±10.7	51.5 ±12.2	115	0.92	Welch
BMI (kg/m²), mean ±SD		24.0 ±2.71	25.5 ±2.61	115	0.022	Welch
Number of sessions, median (Q25-75)		1.00 (1.00; 2.00)	3.00 (3.00; 4.00)	115	<0.001	Mann-Whitney
Size (mm), mean ±SD		8.57 ±1.93)	14.9 ±1.96	115	<0.001	Mann-Whitney
	≤10 mm	8.05 ±1.49)	10.0	83		
	>10 mm	11.8 ±0.899)	15.2 ±1.62	32		
Auxiliary procedures, n (%)	without	95 (100%)	10 (50%)	105	<0.001	Fisher
	ureteral stent	0 (0%)	6 (30%)	6	-	-
	ureteroscopy	0 (0%)	4 (20%)	4	-	-
Complications, n (%)	without	95 (100%)	8 (40%)	103	<0.001	Fisher
	steinstrasse	0 (0%)	4 (20%)	4	-	-
	infection	0 (0%)	4 (20%)	4	-	-
	obstruction	0 (0%)	4 (20%)	4	-	-
Sex, n (%)	male	62 (65%)	13 (65%)	75	0.98	Chi2
	female	33 (35%)	7 (35%)	40	-	-
Side, n (%)	right	48 (51%)	10 (50%)	58	0.97	Chi2
	left	47 (49%)	10 (50%)	57	-	-

**Figure 4:**
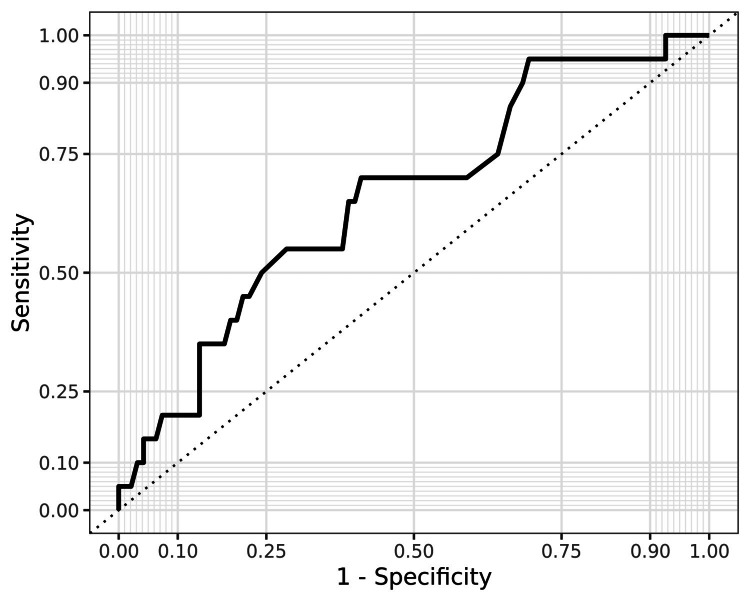
ROC curve—sensitivity and specificity of BMI within non-obese range ROC: Receiver operating characteristic, BMI: Body mass index

Furthermore, logistic regression was performed. Univariate logistic regression analysis showed that BMI, within the range of 18.5-29.9 kg/m², was a statistically significant predictor of ESWL outcome (OR 1.25, 95% CI: 1.04-1.54, p=0.024). When BMI increases by 1 kg/m², the odds of treatment failure are multiplied on average by 1.25. The regression coefficient was 0.277. The area under the ROC curve (AUC) was 0.655 (0.552-0.783) (Figure 4).

Furthermore, univariate regression analysis showed that stone size within the range of 5 mm to 18 mm, was statistically a significant predictor of the treatment outcome (OR 3.84, 95% CI: 2.31-8.97, p=0.001) (Table 3).

**Table 3:** Logistic regression analysis of variables predicting treatment failure CI: Confidence interval, BMI: Body mass index

	Univariate			Multivariate		
	Odds ratio (95% CI)	p-value	Coefficients	Odds ratio (95% CI)	p-value	Coefficients
Size (mm)	3.84 (2.31-8.97)	<0.001	1.35	3.82 (2.30- 8.90)	<0.001	1.34
BMI (kg/m²)	1.25 (1.04-1.54)	0.024	0.277	1.05 (0.714- 1.57)	0.81	0.0459

**Figure 5:**
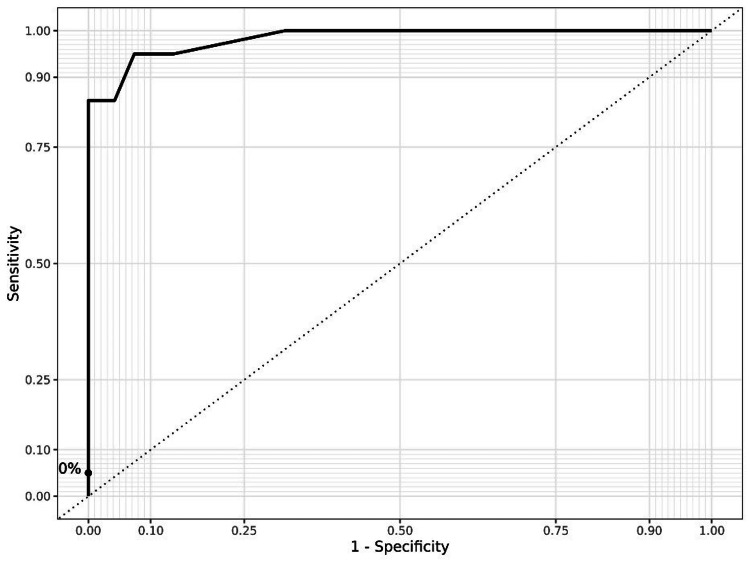
ROC curve–sensitivity and specificity of stone size ROC: Receiver operating characteristic

When stone size increases by 1 mm, the odds of treatment failure are multiplied on average by 3.84. At the threshold of 10 mm sensitivity was 100% and specificity 86%. As the stone size increases, the specificity increases, and the sensitivity decreases (Figure 5). The regression coefficient was 0.277. The AUC was 0.983 (0.968-1.01).

We carried out a multivariate predictive analysis to determine the statistical relationship between treatment failure and stone size by adjusting for BMI. At the risk of 5%, by adjusting for BMI (OR 1.05, 95% CI: 0.714-1.57 p=0.81) there is a statistically significant relationship between treatment outcome and stone size only (OR 3.82, 95% CI: 2.30-8.90, p <0.001) (as seen above in Table 3). The regression coefficient was 1.34. The AUC was 0.985 (0.970-1.01). The other variables introduced in the statistical model are not significantly linked to the outcome.

## Discussion

Urolithiasis is the third most common urological condition and a significant cause of morbidity in 10% to 15% of people worldwide. Dietary and lifestyle changes lead to a predicted increment of almost 2 million lives by the year 2050, with a 25% increase in health care expenses [19]. Ever since 1980, when it was first introduced in the treatment of urolithiasis, ESWL has become an important part of urological practice [5]. It was quickly widely accepted due to being a non-invasive, highly efficient, and cost-effective method. Although the focus of the treatment has slightly shifted toward endourologic procedures in recent times, ESWL remains a preferred modality for certain patients, based on stone size and location [8].

When it comes to ureterolithiasis, up to 70% of stones are located in the distal third of the ureter [8]. Active treatment is indicated if the stone size is >5 mm, with persistent pain resistance to medical therapy, absence of spontaneous stone elimination, and complications such as urinary tract infection, obstructive anuria, etc. According to the American Urological Association (AUA) and European Association of Urology (EAU) guidelines, both URS and ESWL represent the treatment of choice for ureterolithiasis [8,14]. In patients with mid or distal ureteral stones who require intervention, clinicians should recommend URS as first-line therapy. Furthermore, for patients who decline URS, clinicians should offer ESWL [14].

Males are considered to be three times more susceptible to urolithiasis because of testosterone-induced oxalate production and smaller ureteral caliber [5]. Yazici et al. report the proposed 3:1 ratio in their findings, while other studies showed no statistically significant difference regarding sex [20,21]. In our study, 65% of patients were male, and 35% were female, with a male/female ratio of 1.87. Age analysis shows that majority of patients were in the age group 40 to 60 years, which is consistent with previous reports in the literature [10,20,21]. This emphasizes the role of urolithiasis as a significant medical but also socio-economic problem due to its potential to incapacitate the available human resources, combined with the rising cost of treatment.

In terms of the treatment outcome of ESWL, there are several factors involved. Some of them refer to the patient (BMI, SSD, renal function) and urinary tract anatomy (anomalies, obstruction), while others are related to the stone (size, location). Since the ultimate therapeutic goal is to disintegrate the stone and allow its complete clearance, the stone density (measured in HU) is also a notable parameter [22,23]. In our study, the mean stone size was 9.68 ±3.10 mm, with a range from 5 to 18 mm. Other authors also report a similar median stone size of <10 mm in their samples [7]. The EAU guidelines recommend both ESWL and URS for distal ureteral stones <10 mm, but favor URS for stones >10 mm [8]. However, in everyday practice, experienced urologists often make individual decisions based on current guidelines as well as personal expertise and patient preference.

A study published by Yoon et al. examined the correlation between ESWL intensity and the outcome of ureteral stone treatment. Their results also indicate that stone size and density are the key determinants in a successful outcome, not only in the initial session but also in follow-up sessions, while ESWL intensity was not significantly related to the result [20].

After the initial ESWL treatment, stone fragments can be passed over days, weeks, and even months. The need for subsequent treatments or auxiliary treatment options depends on fragment size and associated complications [5]. Treatment outcome was measured as SFR three months after the procedure. We report an overall SFR of 82.6% and an EQ of 0.76. The highest SFR was in the group of patients with stones <10 mm, furthermore establishing a very strong negative correlation between stone size and SFR. In the current study, a single ESWL treatment was sufficient for 59.1% of all patients. In the group of patients with a stone size less than or equal to 10 mm, the success rate after three months was 99%. Patients with stones >10 mm required mostly additional sessions and the success rate after three months was 9.38%. Our analysis showed a statistically significant difference between stone size and the number of sessions only (p <0.001) in the first group and stone size, treatment outcome (p <0.001), and auxiliary procedures (p <0.001) in the second group.

We compared the SFR after the initial ESWL treatment with subsequent sessions. The SFR after the first treatment was 59.1%, 17.4% after the second, and 6.1% after the third session. Cumulative SFR following two sessions was 77%. A low success rate of ESWL retreatment after initial failure was reported by Pace et al. when 1588 patients had an initial SFR of 68%, followed by 46% after the second, and 31% after the third treatment. They reported a cumulative SFR of 77% after the final session [24].

Taking these results into consideration, follow-up ESWL treatment after initial failure should be reconsidered. While there are no definitive guidelines on the number of recommended ESWL treatments per single stone, most authors suggest no more than three, due to previously discussed minimal improvement in cumulative SFR [21,24]. The ultimate decision depends on time to retreatment, patient preference, and overall clinical practice of the institution regarding urolithic procedures.

A meta-analysis that included 1607 patients reports an overall SFR of 73% for distal ureteral stones. With increased stone size, the number of retreatments increased, and SFR decreased [7]. Similar to our results, auxiliary procedures were rarely required, 0.37 per patient.

Initial stone fragmentation shows a predictive value in terms of ESWL success. These fragments usually spontaneously pass to the bladder but sometimes crushed fragments accumulate in the distal ureter, creating steinstrasse [8]. In our study, all these cases were resolved conservatively or by additional ESWL treatment of the leading fragment. We excluded patients taking MET to avoid bias. While MET is common practice in aiding stone passage after ESWL, some authors went beyond and suggested certain lifestyle activities to help pass stone fragments. Li et al. report that sexual intercourse at least three times a week can significantly improve SFR, relieve pain and reduce the formation of steinstrasse after the initial ESWL treatment [25].

We report nine cases of failed ESWL procedures. In six patients there was a failed fragmentation following initial ESWL treatment, while the remaining three had partial fragmentation. All of them required auxiliary procedures to resolve ureteral stones, such as ureteral stents and URS.

Although ESWL is minimally invasive and highly effective, URS provides immediate success, especially with larger stones. However, the Cochrane meta-analysis shows that while URS might be more effective than ESWL in the treatment of distal ureteral stones, in terms of SFR and fewer retreatments needed, it is also associated with more additional problems, such as extended hospital stay, higher complication rate, etc. [9]. Furthermore, there is limited evidence supporting the fact that URS is ultimately more cost-effective than ESWL. Even though it provides almost immediate release, it also requires detailed preoperative preparation, anesthesia, and stenting, which lead to prolonged stays at the hospital and associated complications [26]. A large study from Turkey observed 2836 patients with a single ureteral stone sized 5 mm to 15 mm, out of which 1653 patients (58.3%) had distal ureteral stones. They report a success rate of 88.4% for all distal ureteral stones, and as much as 90.4% for those <10 mm in size [27].

In the current study, complications were observed in 10.4% of patients related to infection and obstruction, while other complications such as hematuria were not analyzed due to the lack of data. They were treated conservatively with the use of ESWL or additional procedures. Treatment complications were significantly different depending on stone size, auxiliary procedures, and the number of sessions as their number and severity increased with increasing values.

Several studies have reported that stone size together with BMI can affect ESWL success rates [8,22,23,28]. The authors found that SFR was significantly lower in obese patients than in normal and overweight patients. Pareek et al. suggested that BMI is a significant predictor of treatment success [22]. This effect is probably related to the SSD, but it could be also due to difficulty in localizing the stone and focusing the shock waves in obese patients [23]. Abdelghany et al. suggested that ESWL could be offered as a primary treatment for patients having distal ureteral stones with a stone length of ≤10 mm, a stone width of <8 mm, and a BMI of <30 kg/m^2^ [28]. Our results showed that a higher BMI favors ESWL failure even in the normal and overweight range (18.5-29.9 kg/m^2^) and that BMI can be a good predictor of treatment outcome.

The time to achieve SFS is a major factor in the evaluation of therapeutic modalities for ureterolithiasis. Peschel et al. report an average of 10 days to achieve SFS in ESWL compared to 1.8 days in URS [29]. Patient satisfaction and quality of life are valuable but sometimes overlooked factors in choosing the right treatment. Pearle et al. report a higher patient satisfaction rate for ESWL compared to URS (96% vs. 89%). Even though the authors also report a better attitude of patients treated with ESWL towards retreatment of the same type (100% vs. 87%), the results did not show statistical significance [30]. A previous study indicated that all patients undergoing URS for stones >5 mm were satisfied, as compared to 95% of those undergoing ESWL [29]. Sonmez et al. concluded that URS without a ureteral stent was the most advantageous technique in terms of patients' daily physical functioning despite the need for postoperative emergency stenting in some cases [31].

While both options represent distinguished and irreplaceable methods in modern urology, the final choice of treatment should be made following patient status, overall health, current guidelines, and institutional and organizational practice. In terms of patient safety, ESWL is a practical, safe and effective method, especially for stones in the distal ureter due to the surrounding anatomy. In absence of parenchymal organs around, shock waves do not cause adjacent tissue damage as in kidney stones and maximum energy can often be delivered.

Furthermore, ESWL could be more practical for hospitals because unlike URS it is usually performed without anesthesia, which reduces overall costs and allows treatment to be performed as an outpatient procedure. This is especially beneficial in the cases of small-size stones <10 mm where a single treatment often provides satisfactory results. All of the above is particularly beneficial in current times considering the COVID-19 pandemic and its implications on patients’ health but also on healthcare system organization and efficacy.

This study included potential weaknesses and limitations because of its retrospective nature and inconsistency in evaluating success by imaging methods: a CT scan was rarely used for the detection of residual fragments after the procedures. Considering the limitations of plain films, the occurrence of residual fragments may be higher as KUB plain radiograph is known to increase the SFR. However, most authors believe that high-quality KUB is usually sufficient to demonstrate the absence of stone material in the ureter, especially in asymptomatic patients [32,33]. Uncritical overuse of CT examinations results in extensive exposure of the patients to radiation and unnecessarily increases the cost of treatment [33].

## Conclusions

Extracorporeal shock wave lithotripsy continues to be a safe and effective option for managing simple calculi in distal ureters with a diameter of ≤10 mm. Treatment outcome was significantly different depending on stone size, BMI, number of treatments, complications, and auxiliary procedures. However, the stone size and BMI, even in the non-obese range, remain significant predictors of treatment outcome. Further studies with larger numbers of cases are needed to determine the parameters of a statistical predictive model for ESWL outcomes.

## References

[1] Ziemba JB, Matlaga BR (2017). Epidemiology and economics of nephrolithiasis. Investig Clin Urol.

[2] Scales CD Jr, Smith AC, Hanley JM, Saigal CS (2012). Prevalence of kidney stones in the United States. Eur Urol.

[3] Cao D, Yang L, Liu L (2014). A comparison of nifedipine and tamsulosin as medical expulsive therapy for the management of lower ureteral stones without ESWL. Sci Rep.

[4] Chand RB, Shah AK, Pant DK, Paudel S (2013). Common site of urinary calculi in kidney, ureter and bladder region. Nepal Med Coll J.

[5] Petrides N, Ismail S, Anjum F, Sriprasad S (2020). How to maximize the efficacy of shockwave lithotripsy. Turk J Urol.

[6] Desai M, Sun Y, Buchholz N (2017). Treatment selection for urolithiasis: percutaneous nephrolithomy, ureteroscopy, shock wave lithotripsy, and active monitoring. World J Urol.

[7] Preminger GM, Tiselius HG, Assimos DG (2007). 2007 guideline for the management of ureteral calculi. J Urol.

[8] Türk C, Neisius A, Petřík A (2021). Urolithiasis - INTRODUCTION - Uroweb. https://uroweb.org/guidelines/urolithiasis.

[9] Aboumarzouk OM, Kata SG, Keeley FX, McClinton S, Nabi G (2012). Extracorporeal shock wave lithotripsy (ESWL) versus ureteroscopic management for ureteric calculi. Cochrane Database Syst Rev.

[10] Yang C, Li S, Cui Y (2017). Comparison of YAG laser lithotripsy and extracorporeal shock wave lithotripsy in treatment of ureteral calculi: a meta-analysis. Urol Int.

[11] COVIDSurg Collaborative (2020). Mortality and pulmonary complications in patients undergoing surgery with perioperative SARS-CoV-2 infection: an international cohort study. Lancet.

[12] Pishchalnikov YA, Sapozhnikov OA, Bailey MR (2003). Cavitation bubble cluster activity in the breakage of kidney stones by lithotripter shockwaves. J Endourol.

[13] Alizadeh M, Magsudi M (2014). The effect of tamsulosin in the medical treatment of distal ureteral stones. Glob J Health Sci.

[14] Assimos D, Krambeck A, Miller NL (2016). Surgical management of stones: American Urological Association/Endourological Society Guideline, PART I. J Urol.

[15] Kang DH, Cho KS, Ham WS, Lee H, Kwon JK, Choi YD, Lee JY (2016). Comparison of high, intermediate, and low frequency shock wave lithotripsy for urinary tract stone disease: systematic review and network meta-analysis. PLoS One.

[16] Falahatkar S, Khosropanah I, Vajary AD, Bateni ZH, Khosropanah D, Allahkhah A (2011). Is there a role for tamsulosin after shock wave lithotripsy in the treatment of renal and ureteral calculi?. J Endourol.

[17] Elkoushy MA, Hassan JA, Morehouse DD, Anidjar M, Andonian S (2011). Factors determining stone-free rate in shock wave lithotripsy using standard focus of Storz Modulith SLX-F2 lithotripter. Urology.

[18] Denstedt J, Clayman RV, Preminger G (1990). Efficiency quotient as a means of comparing lithotripters. J Endourol.

[19] Sorokin I, Mamoulakis C, Miyazawa K, Rodgers A, Talati J, Lotan Y (2017). Epidemiology of stone disease across the world. World J Urol.

[20] Yazici O, Tuncer M, Sahin C, Demirkol MK, Kafkasli A, Sarica K (2015). Shock wave lithotripsy in ureteral stones: evaluation of patient and stone related predictive factors. Int Braz J Urol.

[21] Yoon JH, Park S, Kim SC, Park S, Moon KH, Cheon SH, Kwon T (2021). Outcomes of extracorporeal shock wave lithotripsy for ureteral stones according to ESWL intensity. Transl Androl Urol.

[22] Pareek G, Armenakas NA, Panagopoulos G, Bruno JJ, Fracchia JA (2005). Extracorporeal shock wave lithotripsy success based on body mass index and Hounsfield units. Urology.

[23] Park BH, Choi H, Kim JB, Chang YS (2012). Analyzing the effect of distance from skin to stone by computed tomography scan on the extracorporeal shock wave lithotripsy stone-free rate of renal stones. Korean J Urol.

[24] Pace KT, Weir MJ, Tariq N, Honey RJ (2000). Low success rate of repeat shock wave lithotripsy for ureteral stones after failed initial treatment. J Urol.

[25] Li W, Mao Y, Lu C, Gu Y, Gu X, Hua B, Pan W (2020). Role of sexual intercourse after shockwave lithotripsy for distal ureteral stones: a randomized controlled trial. Urol J.

[26] Shah OD, Matlaga BR, Assimos DG (2003). Selecting treatment for distal ureteral calculi: shock wave lithotripsy versus ureteroscopy. Rev Urol.

[27] Demirbas M, Samli M, Karalar M, Kose AC (2012). Extracorporeal shockwave lithotripsy for ureteral stones: twelve years of experience with 2836 patients at a single center. Urol J.

[28] Abdelghany M, Zaher T, El Halaby R, Osman T (2011). Extracorporeal shock wave lithotripsy of lower ureteric stones: outcome and criteria for success. Arab J Urol.

[29] Peschel R, Janetschek G, Bartsch G (1999). Extracorporeal shock wave lithotripsy versus ureteroscopy for distal ureteral calculi: a prospective randomized study. J Urol.

[30] Pearle MS, Nadler R, Bercowsky E (2001). Prospective randomized trial comparing shock wave lithotripsy and ureteroscopy for management of distal ureteral calculi. J Urol.

[31] Sonmez G, Demir F, Keske M, Karadag MA, Demirtas A (2021). Comparison of the effects of four treatment techniques commonly used in ureteral stone treatment on patients' daily physical functioning: an observational randomized-controlled study. J Endourol.

[32] Chaussy CG, Tiselius HG (2018). How can and should we optimize extracorporeal shockwave lithotripsy?. Urolithiasis.

[33] Hussein A, Anwar A, Abol-Nasr M, Ramadan E, Shaaban A (2014). The role of plain radiography in predicting renal stone fragmentation by shockwave lithotripsy in the era of noncontrast multidetector computed tomography. J Endourol.

